# Shear Bond Strength in Stone-Clad Façades: Effect of Polypropylene Fibers, Curing, and Mechanical Anchorage

**DOI:** 10.3390/polym16212975

**Published:** 2024-10-24

**Authors:** Vahid Shafaie, Oveys Ghodousian, Amin Ghodousian, Mohammad Gorji, Hossein Mehdikhani, Majid Movahedi Rad

**Affiliations:** 1 Department of Structural and Geotechnical Engineering, Széchenyi István University, 9026 Győr, Hungary; shafaie.vahid@sze.hu (V.S.); majidmr@sze.hu (M.M.R.); 2 Department of Civil Engineering, Takestan Branch, Islamic Azad University, Takestan 3481949479, Iran; m_gorji60@yahoo.com (M.G.);; 3 Department of Engineering Science, University of Tehran, Tehran 1417935840, Iran; a.ghodousian@ut.ac.ir

**Keywords:** shear bond strength, façade stones, mechanical anchorage, polypropylene fibers, fuzzy logic prediction

## Abstract

This study investigates the shear bond strength between four widely used façade stones—travertine, granite, marble, and crystalline marble—and concrete substrates, with a particular focus on the role of polypropylene fibers in adhesive mortars. The research evaluates the effects of curing duration, fiber dosage, and mechanical anchorage on bond strength. Results demonstrate that Z-type anchorage provided the highest bond strength, followed by butterfly-type and wire tie systems. Extended curing had a significant impact on bond strength for specimens without anchorage, particularly for travertine. The incorporation of polypropylene fibers at 0.2% volume in adhesive mortar yielded the strongest bond, although lower and higher dosages also positively impacted the bonding. Furthermore, the study introduces a novel fuzzy logic model using the Dombi family of t-norms, which outperformed linear regression in predicting bond strength, achieving an R^2^ of up to 0.9584. This research emphasizes the importance of optimizing fiber dosage in adhesive mortars. It proposes an advanced predictive model that could enhance the design and safety of stone-clad façades, offering valuable insights for future applications in construction materials.

## 1. Introduction

The aesthetics of structures have long captivated interest, with building façades serving as a defining feature that enhances both the visual appeal and integrity of urban environments. The selection of façade materials—encompassing stone, brick, glass, composites, and white cement—is influenced not only by aesthetic considerations but also by durability, availability, cost, and resilience to environmental factors. Among these materials, natural stones such as granite, marble, and travertine are particularly favored for their durability and visual allure. However, the prolonged exposure of stone façades to environmental conditions raises concerns about detachment, which can result in significant safety hazards. This underscores the critical importance of ensuring the secure bonding of façade stones to mitigate such risks. Granite, with its inherent density and exceptional resistance to moisture and impact, is a highly regarded option, although its processing costs are considerable. Marble, while more cost-effective and easier to work with, is notably less resistant to weathering. Travertine, characterized by its porous structure, presents additional challenges due to its high water permeability. Despite these complexities, natural stone remains an indispensable material in façade design, with ongoing research addressing both performance and safety considerations.

The mechanical performance and durability of stone cladding systems have been widely studied, with particular emphasis on the role of adhesive and anchorage methods. Research has shown that the inclusion of adhesive significantly enhances the ultimate load capacity of anchorage systems and influences their failure characteristics [1]. Sustainable design guidelines for efficient façade cladding systems in hot climates have been developed, with a focus on environmental performance [2]. Public safety concerns, particularly the risk of falling objects from aging high-rise façades, have also been raised [3]. Studies have further demonstrated the effect of curing duration and the addition of an anti-freezing agent in cement–sand mortar, which improves the bond strength between façade stones and concrete substrates [4]. Additionally, the performance of natural stone façades under various environmental and structural conditions has been analyzed. Laboratory and field tests on different anchorage systems applied to façade stones have been conducted [5]. In contrast, methods for maintaining the durability of façade stones under diverse environmental conditions have been discussed [6]. Seismic performance evaluations of anchored stone panels subjected to direct shear and cyclic loading have also been carried out [7]. A methodology using the Factor Method has been developed to predict the service life of natural stone façades under environmental stress [8]. Furthermore, the effects of high temperatures and thermal shock on anchored granite façades have been examined [9], and investigations on historical masonry façades have utilized pull-off and porosity tests, mineralogical analyses, and thermography to assess their integrity [10]. These studies collectively underscore the critical relationship between material properties, environmental exposure, and anchorage methods in ensuring the long-term performance and safety of stone façade systems.

In building construction, the use of anchorage systems is indispensable for securely fastening stone façades to their substrates, particularly due to the challenges posed by the smooth surface and low permeability of many stone types. Reliance on adhesive attachment alone is often insufficient, especially for vertical installations, where the risk of stone detachment under seismic forces or heavy wind loads is a concern. Mechanical anchorage, including wire ties, butterfly-type clips, and Z-type clips, offers a reliable solution for enhancing the stability and durability of stone cladding. Given that adhesive attachment is generally restricted to lighter stone units due to safety standards, heavier or larger stones require mechanical anchorage to ensure structural integrity [11]. This is critical for preventing stone displacement and potential hazards, particularly in high-rise or high-stress environments.

The splitting method has been selected for evaluating the bond strength between façade stones and their concrete substrates, primarily due to its established effectiveness in concrete-to-concrete bonding assessments [12,13,14,15,16]. Although this technique has been widely applied in concrete bonding tests, its use in measuring the bond strength between façade stones and their substrates has been relatively limited. Given its successful application in determining bond strength in old-to-new concrete composites, this method has been adopted in the present study for façade-stone bonding. The technique subjects a square cross-section test specimen to longitudinal shear loading, leading to failure along the plane intersecting the load axis, thereby splitting the specimen into two parts [17]. The bond strength is ultimately calculated by dividing the applied force by the cross-sectional area of the specimen.

Fiber reinforcement is commonly used in concrete or mortar applications to improve performance in tunnel linings, pavements, and repair overlays. The addition of fiber reduces plastic shrinkage in fresh concrete [18] and increases permeability, toughness, and fatigue resistance in the hardened material [19,20]. Polypropylene fibers have attracted significant attention due to their beneficial effects. The influence of polypropylene fibers on the rheological properties and compressive strength of self-compacting concrete under prolonged mixing has been studied [21], as well as their effect on in situ strength [22]. However, research specifically exploring the impact of polypropylene fibers on the behavior of adhesive mortar behind façade stones remains lacking.

Fuzzy logic systems have been increasingly employed in recent years to predict various concrete properties across a wide range of studies [23,24,25,26,27,28,29]. These systems have demonstrated effectiveness in calculating interfacial bond strength in self-compacting concrete repair layers through the use of the GNNC Modified PSO algorithm and predicted the 28-day compressive strength of pozzolanic concrete [30,31]. Additionally, predictive models incorporating fuzzy logic have been applied to forecast concrete shrinkage, using a combination of fuzzy systems and genetic algorithms [32] illustrated by an in-depth analysis of colored self-compacting concrete using multi-objective optimization through genetic algorithms [33]. Furthermore, Mamdani-type fuzzy logic models have been developed to assess bond behavior in lightweight concrete [34], and fuzzy logic techniques have successfully been applied to estimate engineering properties in pre-placed aggregate concrete [35]. In other applications, fuzzy logic models have predicted compressive strength in concrete containing green foundry sand [36] and the mechanical properties of blended cement at elevated temperatures [37]. The bond strength between self-compacting mortar and normal vibrated concrete has also been modeled using fuzzy logic systems [26]. Despite the extensive use of fuzzy logic in predicting concrete behavior, there remains a significant gap in the literature concerning its application to the bond strength of façade stones to their substrates and the variables influencing this bond.

This study addresses the crucial topic of bonding between four widely used façade stones—travertine, granite, marble, and crystalline marble—and their concrete substrate, focusing on three key influencing factors: curing duration, polypropylene fiber dosage in adhesive mortars, and the type of mechanical anchorage. The primary objective was to systematically evaluate the effects of these variables on the shear bond strength, providing insights that could enhance the performance and durability of stone-clad façades. A novel contribution of this research lies in the development of a predictive model using a fuzzy logic system, integrating a generalized Mamdani interface and Dombi family t-norms. This approach offers a pioneering method to accurately predict bond strength, thereby advancing both theoretical understanding and practical applications in the field of façade engineering.

## 2. Materials and Methods

### 2.1. Research Methodology Outline

This study evaluates the bond strength of various façade stones when anchored to a concrete substrate, forming a Composite Specimen consisting of three main components: an unyielding concrete support, an overlay of mortar, and the façade stone. The investigation explores different anchoring systems, types of façade stones, polypropylene fiber concentrations within the mortar overlay, and the effects of various curing periods on the bond strength.

**Figure 1:**
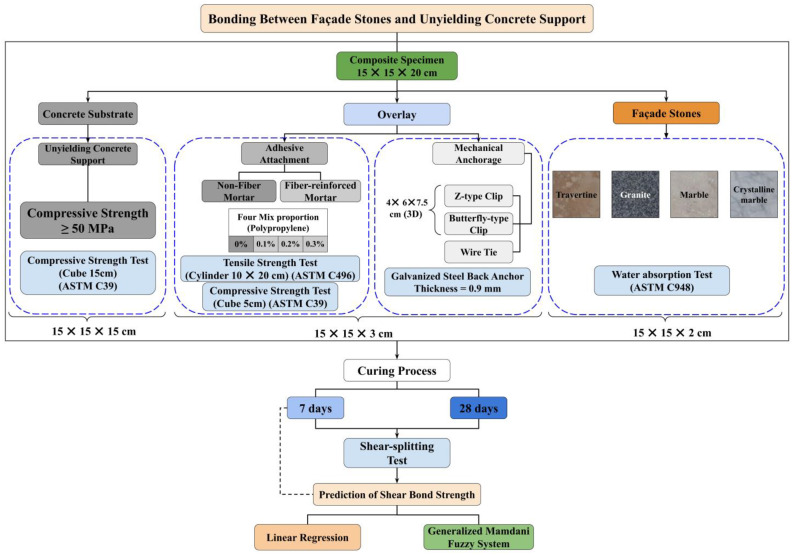
Research flowchart: experimental design and predictive modeling.

The overall research approach is illustrated in Figure 1, which outlines both the experimental design and the predictive modeling process. The experimental design isolates the impact of each variable—composite specimen configuration, anchoring methods, façade stone type, and curing duration—on bond strength. Following the experimental phase, predictive models are employed to estimate shear bond strength using linear regression and a Generalized Mamdani Fuzzy System based solely on data from the 7-day curing process.

### 2.2. Material Selection and Preparation

The composite specimens in this study consist of three primary components: unyielding concrete supports, an overlay, and façade stones.

#### 2.2.1. Unyielding Concrete Support

The concrete supports were designed as 15 cm^3^ cubes with a compressive strength of not below 50 MPa, ensuring they would provide a stable and unyielding base for the overlay and façade stones. The term “Unyielding” was selected to highlight the high compressive strength of these supports, ensuring minimal deformation under load.

#### 2.2.2. Overlay

**Figure 2:**
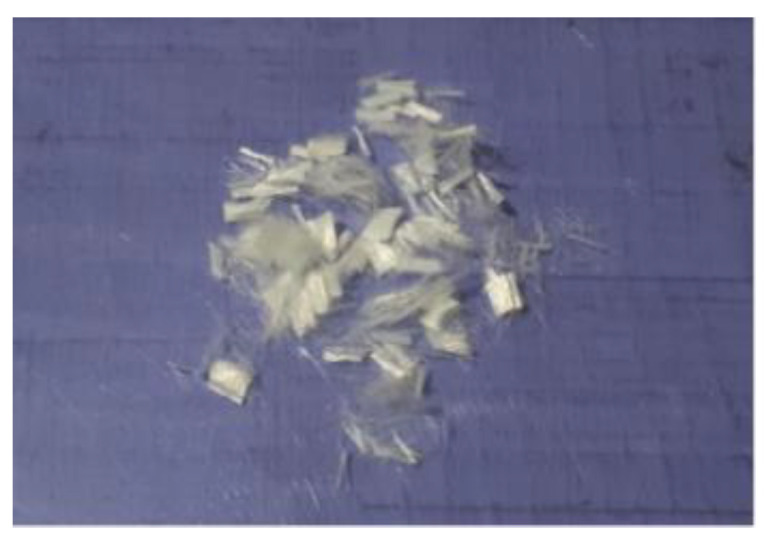
The polypropylene fibers.

The overlay, positioned between the concrete support and the façade stones, was divided into two functional categories:

**Adhesive Attachment:** Adhesive mortars were prepared using four different mix proportions, incorporating polypropylene fibers in varying dosages of 0%, 0.1%, 0.2%, and 0.3% by volume of mortar (Vm). The polypropylene fibers, with a length of 6 mm, a diameter of 200 μm, and a density of 0.91 g/cm^3^ (Table 1), were included to assess their impact on bonding efficacy and to enhance bond strength (Figure 2). The dispersion of polymer fibers within the adhesive mortar presents significant challenges due to the inherent hydrophobicity and non-polar characteristics of polypropylene. However, in this study, the volume of adhesive mortar used was minimal, and it was prepared directly at the site of application, eliminating issues related to transportation that could affect fiber dispersion. Additionally, meticulous attention was given to the addition of polypropylene fibers to the mortar to ensure even distribution and avoid concentration at any single point. Multiple visual inspections confirmed that the fibers were uniformly distributed throughout the adhesive mortar. The mix proportions for each adhesive mortar are detailed in Table 2.

All mixes maintained a water-to-cement (W/C) ratio of 0.5 to ensure uniform consistency across specimens. For consistency in all mortar mixes, Cement type CEMI 42.5 (a Portland cement with a 28-day compressive strength of 42.5 MPa) was selected, combined with rounded fine aggregate having a maximum particle size of 4.75 mm, a density of 2700 kg/m^3^, and a 24 h water absorption rate of 1.5% [38]. These materials were chosen to ensure uniformity and to maintain the integrity of the composite specimens throughout testing.

**Figure 3:**
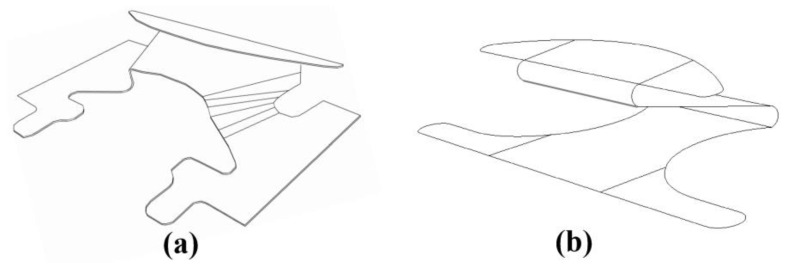
Schematic back anchor used in the study: (**a**) butterfly-type clip, (**b**) -type clip.

**Mechanical Anchorage:** To further investigate the bond strength, a subset of specimens utilized mechanical anchorages in combination with adhesive mortars. Three types of back anchors were employed: Z-type clips, butterfly-type clips (Figure 3), and wire ties (depicted in the installation process in the following subsection, Figure 5). These anchors were specifically selected for their ability to enhance the mechanical interlock between the stone and the substrate, improving resistance to shear stresses.

The butterfly-type and Z-type clips were fabricated from galvanized steel Grade 37 (ultimate stress of 370 MPa) with a thickness of 0.9 mm and dimensions of 4 × 6 × 7.5 (cm). These clips are 3D anchors designed to provide enhanced stability through their specific shapes, improving their mechanical performance when subjected to load. The wire ties were used as an additional form of mechanical anchoring, attached at the back of the stone façade.

The material properties of the galvanized steel, including yield stress, ultimate stress, and ultimate strain, are presented in Table 3 below.

#### 2.2.3. Façade Stones

**Figure 4:**
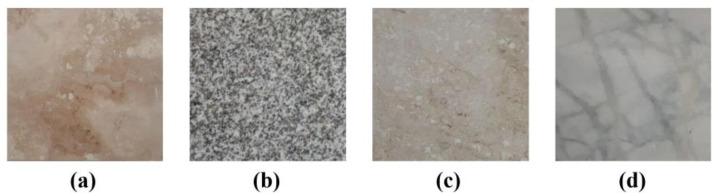
Façade stones used in the study: (**a**) travertine, (**b**) granite, (**c**) marble, (**d**) crystalline marble.

Four types of façade stones commonly used in the construction industry were selected: travertine, granite, marble, and crystalline marble. These stones were precisely cut to dimensions of 15 × 15 × 2 (cm) to ensure consistent bonding with the composite specimens (Figure 4).

### 2.3. Test Setup and Protocols

#### 2.3.1. Anchoring and Bonding Procedure

**Figure 5:**
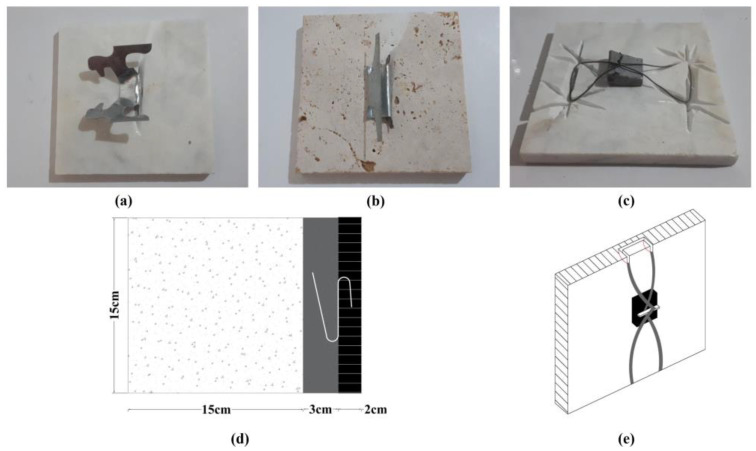
Anchorage installations on façade stones: (**a**) butterfly-type clip, (**b**) Z-type clip, (**c**) wire tie, (**d**) schematic of composite specimen showing three layers: 15-cm concrete substrate, 3-cm overlay of adhesive mortar, and 2-cm façade stone, with schematic anchorage placement, (**e**) schematic installation of wire tie.

In wall cladding systems, particularly for façade installations, back anchoring methods are commonly used to secure façade stones to the concrete substrate. In this study, butterfly-type clips, Z-type clips, and wire ties were selected for their ability to provide mechanical interlock. The anchorage system was first attached to the façade stones, with one part of the anchor embedded in the stone and the other part positioned within the adhesive mortar. This configuration connected the façade stone to the unyielding concrete support, ensuring stability and load transfer. The full installation process, including the bonding of the anchorages to the façade stones, is illustrated in Figure 5.

**Figure 6:**
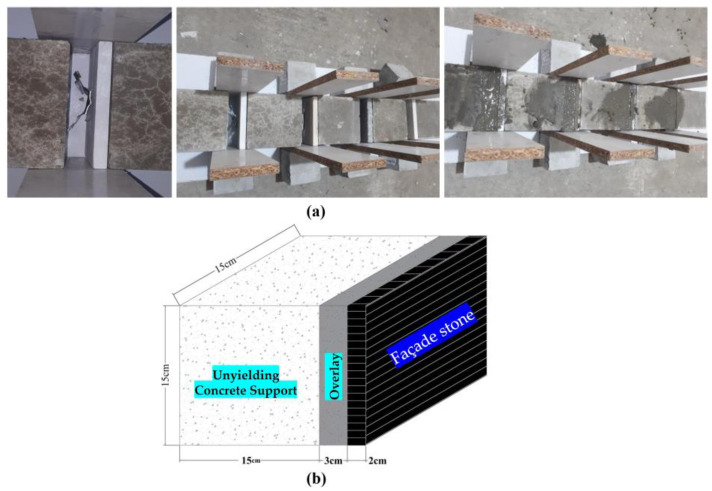
Preparation of composite specimens; (**a**) laboratory preparation process: left—placement of anchorage, middle—molding of the specimen, right—application of adhesive mortar; (**b**) schematic representation of the final assembly.

Following the installation of the anchorages, cement–sand mortars, incorporating both fiber-reinforced and non-fiber variants, were applied to bond the façade stones to the 15 cm cubic concrete substrates. To create the composite specimens, the unyielding concrete support (15 × 15 × 15 (cm)) was positioned, and a 2 cm-thick façade stone was attached, leaving a 3 cm gap between the concrete substrate and the façade stone to accommodate the overlay mortar as the adhesive layer, effectively forming a mold for the mortar. The mortar casting process marked the final stage in the production of the composite specimens, resulting in a total specimen thickness of 20 cm. The procedure for the application of adhesive mortars and the creation of the composite specimens is illustrated in Figure 6. Figure 6a includes three stages: left—the method of placing the anchorage on the surface of the façade stone; middle—the molding of the specimen before applying the adhesive mortar; and right—the moment after applying the adhesive mortar between the stone and the concrete substrate. Figure 6b provides a schematic representation of the composite specimen, showing the dimensions of each layer in detail.

#### 2.3.2. Physical Testing of Façade Stones and Adhesive Mortar

The 24 h water absorption rates of the selected façade stones—travertine, granite, marble, and crystalline marble—were determined according to the guidelines of [39]. This test was crucial for understanding the porosity and moisture retention capacity of each stone type, which can directly impact the bond performance in wall cladding applications. To prevent the stones from absorbing moisture from the adhesive during bonding, each stone was pre-saturated by submersion in water for 24 h. This pre-saturation ensured consistent curing of the mortar and reduced the risk of improper adhesive bonding due to moisture absorption.

**Figure 7:**
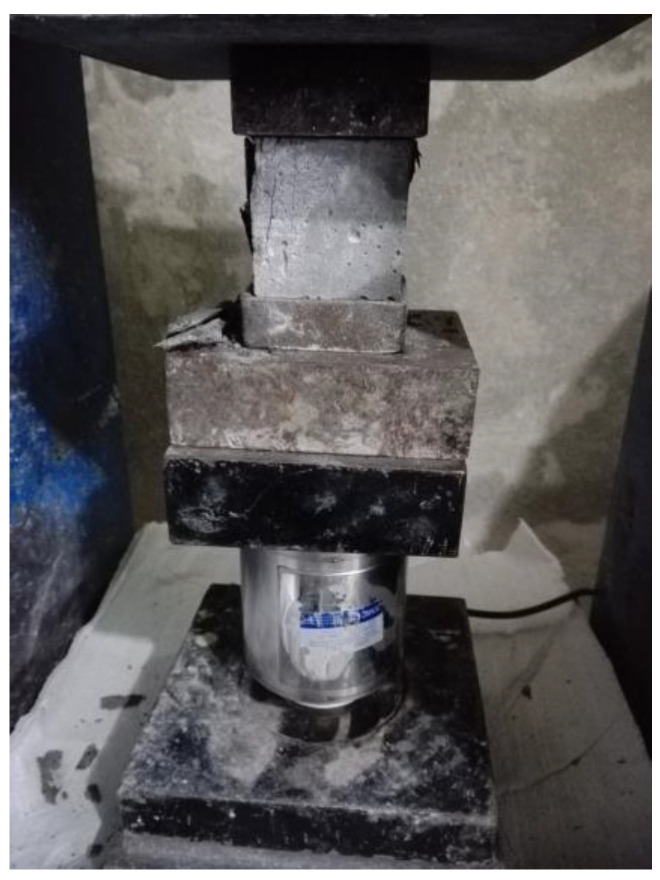
Compressive strength test setup for 5 cm cubic adhesive mortar specimens.

For the adhesive mortars, compressive strength tests were conducted on 5 cm cubic specimens, while tensile strength tests (Brazilian method) were performed on cylindrical specimens with a diameter of 10 cm and a height of 20 cm, following [40,41]. This test evaluated the mechanical properties of the mortars used in bonding the façade stones to the unyielding concrete substrate. By measuring the mortar’s resistance to compression, this assessment ensured the integrity of the adhesive layer under the expected structural loads in practical applications. The testing procedure and setup are depicted in Figure 7, highlighting the critical role of mortar strength in the composite specimens’ overall performance. It is worth mentioning that in each case, three specimens were tested, and the average of the three tests was reported as the final result for each test in the paper.

#### 2.3.3. Bond Strength Evaluation

The shear bond strength of the composite specimens was evaluated using a shear splitting test, a crucial method for assessing the adhesion between the façade stones and the adhesive mortars. Prior to testing, the specimens were subjected to a water-curing process for either 7 or 28 days to ensure consistent hydration. The test setup applied lateral loading to the specimens, simulating the stresses experienced in real-world wall cladding systems until failure occurred at the adhesive interface.

**Figure 8:**
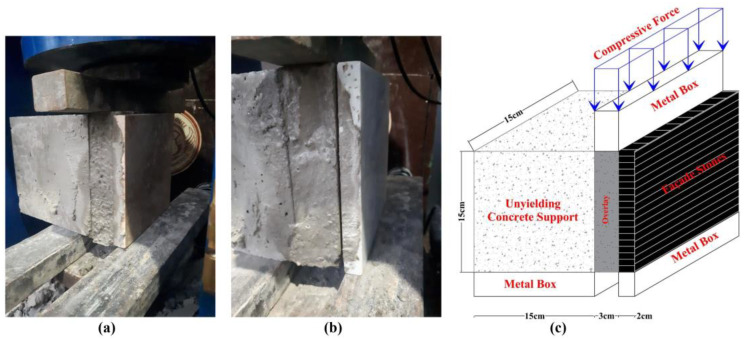
Shear splitting test: (**a**) composite specimen before failure, (**b**) composite specimen after failure at the adhesive interface, and (**c**) schematic representation of the shear splitting test setup.

In this test, a vertical compressive force was applied to the composite specimen, which consisted of a 15 × 15 × 15 (cm) unyielding concrete support, a 3 cm-thick adhesive mortar layer, and a 2 cm-thick façade stone. The load was transmitted through a metal box to ensure uniform pressure distribution across the adhesive interface (Figure 8). The test aimed to simulate the mechanical stresses encountered by façade cladding systems in practical applications.

The failure typically occurred at the weakest interface, either between the façade stone and the adhesive mortar or between the mortar and the concrete support, thereby allowing the precise determination of the maximum load at failure. The results provided critical insights into the bond strength under various conditions, including different anchorage systems, mortar compositions, and fiber dosages. This evaluation is essential for determining the long-term durability and mechanical performance of façade cladding systems. The testing configuration is depicted in Figure 8. The width of the metal box used to transmit the jack force to the specimen was considered equal to the thickness of the adhesive mortar, which is 3 cm, to ensure that the results have sufficient accuracy.

## 3. Results and Discussion

### 3.1. Water Absorption of Façade Stones

The results of the 24 h water absorption test are shown in Table 4. This test evaluated the porosity and moisture retention characteristics of the four façade stones—travertine, granite, marble, and crystalline marble—to assess their potential impact on adhesive attachment with the mortar.

Travertine exhibited the highest absorption rate at 1.36%, indicating its porous nature, which can enhance its interaction with the adhesive mortar. Granite, with the lowest absorption rate of 0.32%, is less porous, which might limit its adhesive attachment potential, but the pre-saturation step ensured moisture absorption from the mortar was prevented. Marble and crystalline marble showed absorption rates of 1.12% and 0.76%, respectively, suggesting moderate porosity. These absorption rates highlight the varying interactions of the stones with the mortar, where higher porosity stones like travertine may promote stronger adhesive attachment, while lower porosity stones like granite might rely more on surface adhesion. The test results provide a clear understanding of how water absorption could influence bonding, laying the foundation for further evaluation through shear bond strength testing.

### 3.2. Mechanical Properties of Adhesive Mortars

The mechanical properties of the adhesive mortars, specifically compressive strength and tensile strength, were evaluated after 7 and 28 days of curing, presented in Table 5. The compressive strength tests were conducted on 5 cm cubic specimens, while the tensile strength measurements were performed according to standard procedures for adhesive mortars. The results are presented in Table 5, demonstrating the influence of varying polypropylene fiber dosages (0%, 0.1%, 0.2%, and 0.3%) on these properties.

After 7 days, the control mix (Ctrl) achieved a compressive strength of 17.43 MPa. Fiber-reinforced mortars showed a modest improvement, with P2 (0.2% fiber) attaining the highest strength of 18.05 MPa, marking a slight increase over the control. Interestingly, the mix with the highest fiber content, P3, displayed a reduction to 16.42 MPa, suggesting that excessive fiber dosage may introduce micro-voids or distribution issues that inhibit early strength development. The results at 28 days exhibited a more consistent pattern of strength improvement across all mixes. The control mix increased to 24.88 MPa, while P2 again demonstrated superior performance at 26.52 MPa, indicating that the moderate fiber dosage contributed to sustained strength gains. However, the P3 mix, despite its fiber reinforcement, reached only 25.28 MPa, further supporting the observation that higher fiber content can negatively impact the long-term compressive strength due to potential compaction issues.

The tensile strength results provide further insight into the role of polypropylene fibers in enhancing the mortar’s resistance to tensile forces. The addition of fibers helps bridge micro-cracks within the mortar matrix, improving the material’s ability to resist tensile stress. This effect is particularly evident in mix P2, which achieved the highest tensile strength of 2.58 MPa at 7 days and 3.86 MPa at 28 days. The presence of fibers mitigates crack propagation under tensile loading, contributing to a more ductile behavior and greater durability of the adhesive mortar. In contrast, the P3 mix, despite its higher fiber content, exhibited a relatively lower tensile strength, potentially due to challenges in maintaining uniform fiber dispersion, which may result in localized weaknesses within the matrix.

Overall, the inclusion of polypropylene fibers positively influences both the compressive and tensile properties of the adhesive mortars, although the extent of improvement varies. Mix P2 (0.2% fiber) was found to be the most effective, providing a balanced enhancement in both compressive and tensile strengths. This dosage appears optimal for improving structural integrity without introducing the drawbacks associated with higher fiber content. In contrast, the P3 mix, with its higher fiber dosage, showed diminishing returns in both compressive and tensile performance, highlighting the need for careful control of fiber content to ensure optimal mechanical properties. These findings suggest that moderate fiber dosages can effectively improve the overall performance of adhesive mortars, but excessive fiber content should be avoided to maintain uniformity and prevent adverse effects on strength characteristics.

### 3.3. Shear Bond Strength Evaluation

**Figure 9:**
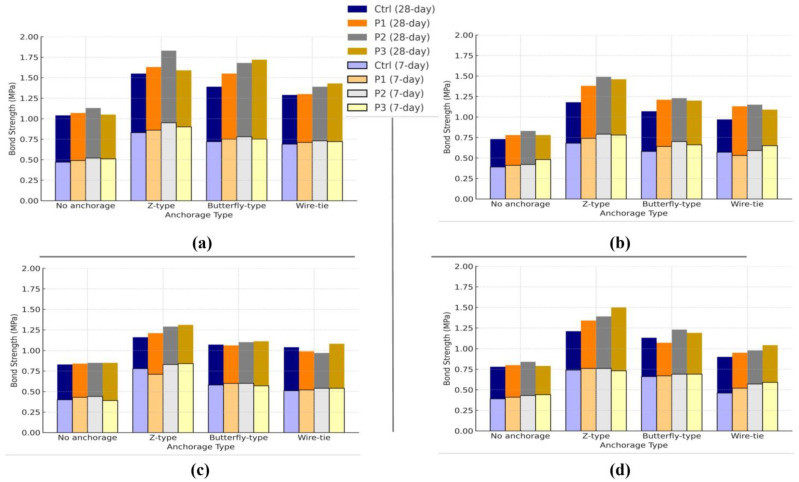
Shear bond strength between façade stones and concrete substrate for (**a**) travertine, (**b**) granite, (**c**) marble, and (**d**) crystalline marble after 7-day and 28-day curing periods with varying polypropylene fiber content (Ctrl, P1, P2, P3) and anchorage types (No anchorage, Z-type clip, butterfly-type clip, and wire tie).

The shear bond strength between the façade stones and the concrete substrate was evaluated using a shear splitting test. The specimens were tested under four anchorage configurations: Z-type clips, butterfly-type clips, wire ties, and no anchorage. The cement–sand adhesive mortar was reinforced with varying dosages of polypropylene fibers: 0%, 0.1%, 0.2%, and 0.3% by volume. The façade stones tested included travertine, granite, marble, and crystalline marble. Bond strength measurements were taken after 7 and 28 days of curing, and the results are presented in Figure 9a–d.

#### 3.3.1. Shear Bond Strength Across Different Façade Stones

The shear bond strength results for travertine, granite, marble, and crystalline marble show consistent trends across the different polypropylene fiber dosages, types of anchorage, and curing periods. Across all stone types, the Z-type clip consistently provided the highest shear bond strength, while specimens without anchorage exhibited the lowest strength, underscoring the essential role of mechanical interlock in enhancing the shear bond performance between the mortar and stone.

For travertine (Figure 9a), the maximum shear bond strength of 1.83 MPa was observed with Z-type clip anchorage and P2 mortar after 28 days of curing, compared to 1.04 MPa for the unanchored specimens using Ctrl mortar. Travertine, with its high water absorption rate (1.36%), benefits from its porous structure, which enhances the penetration and bonding ability of the adhesive mortar. This characteristic makes travertine the top-performing stone in terms of shear bond strength across all anchorage types. In the case of granite (Figure 9b), which has the lowest water absorption (0.32%), the shear bond strength results were lower than travertine, but not the lowest among the four stones. Granite’s maximum shear bond strength was recorded at 1.49 MPa after 28 days with Z-type clip anchorage and P2 mortar, showing the stone’s lower ability to bond well due to its dense structure, which restricts the adhesive interaction between the mortar and the stone. For marble (Figure 9c), the results follow a similar pattern, though marble’s moderate water absorption (1.12%) allows for better bonding than granite, but lower than travertine. The highest shear bond strength of 1.31 MPa was observed with Z-type clip anchorage and P3 mortar after 28 days of curing. Marble’s dense microstructure limits the degree of adhesive interaction compared to travertine, leading to lower overall shear bond strength. Finally, crystalline marble (Figure 9d), with a water absorption rate of 0.76%, displayed shear bond strengths slightly higher than granite but lower than travertine. The maximum shear bond strength for crystalline marble was 1.50 MPa with Z-type clip anchorage and P3 mortar after 28 days of curing. This result highlights the role of moderate porosity in facilitating adhesive interaction, although it does not attain the bond strength observed in more porous stones, such as travertine.

To better understand the effect of stone type on shear bond strength, the results were averaged across all polypropylene fiber dosages, anchorage types, and curing periods. The averaged values are as follows: travertine achieved the highest average shear bond strength at 0.71 MPa, followed by granite at 0.60 MPa, crystalline marble at 0.59 MPa, and marble at 0.58 MPa. These results align with the water absorption characteristics of each stone: travertine’s higher porosity leads to stronger bonding performance, while granite’s dense structure and low water absorption result in comparatively lower bond strength. In conclusion, the type of stone plays a significant role in determining shear bond strength, with travertine consistently outperforming the others due to its porous nature and higher water absorption. Granite and crystalline marble, being denser and less permeable, showed lower bonding performance, while marble, though slightly more porous, still fell behind travertine in terms of overall bond strength. In fact, travertine is the only stone that exhibits significantly higher bond strength compared to the other stones, while the other three stones show relatively similar results. Despite its higher water absorption compared to granite and crystalline marble, marble demonstrates lower bond strength with the adhesive mortar due to its denser texture, which limits the mechanical interlocking between the stone and the adhesive layer.

#### 3.3.2. Effect of Anchorage Type on Shear Bond Strength

**Figure 10:**
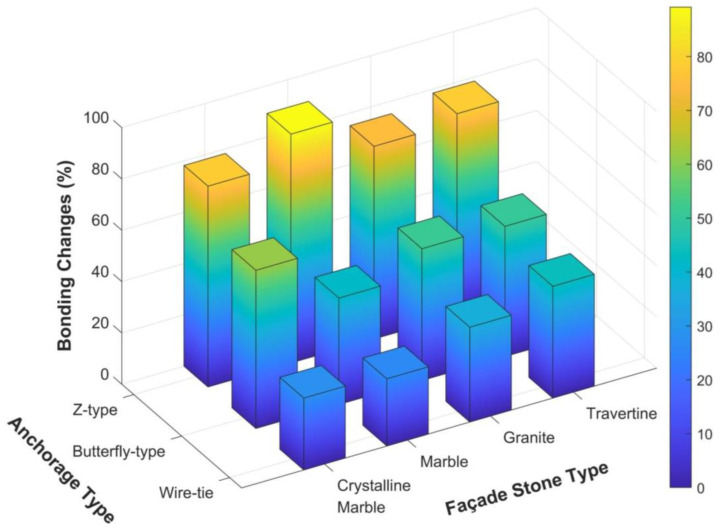
Bond strength growth between façade stones and concrete substrates due to the presence of different types of anchorage systems (Z-type, butterfly-type, and wire tie), compared to the no-anchorage condition. The graph reflects the results based on 7-day curing specimens for various façade stones, including travertine, granite, marble, and crystalline marble.

Figure 10 illustrates the percentage increase in bond strength between the façade stones and the concrete substrate as influenced by different anchorage systems. The analysis focuses on 7-day cured specimens, as this curing period typically meets the standard requirements for adhesive mortars. The objective of this section is to assess how different mechanical anchoring methods—Z-type, butterfly-type, and wire tie—affect bond strength compared to the no-anchorage condition.

It is evident from the figure that the Z-type clip provides the highest bond strength increase across all stone types, with improvements ranging from 75% to 89%. This significant enhancement can be attributed to the uniform distribution of shear stresses across the adhesive interface, particularly benefiting stones like marble, which exhibited a peak improvement of 89.18%. The effectiveness of the Z-type clip can be linked to its design, which facilitates greater mechanical interlocking and adhesion between the stone and the substrate. With the installation of the Z-type clip anchorage on the specimens, the denser texture of the marble allowed for a stronger connection between the anchorage and the stone. The Z-type clip provides a more effective mechanical interlocking with the façade stone compared to other anchorage types, such as the butterfly-type clip and wire tie. This enhanced interlocking is particularly beneficial for denser stones such as marble, as it allows the anchorage to better engage with the stone’s surface, resulting in a significant improvement in bond strength. In contrast, the Butterfly-type clip and Wire tie did not achieve the same level of interlocking, leading to less pronounced improvements in bond strength for marble.

The butterfly-type clip, while slightly less effective than the Z-type, still demonstrates substantial improvements in bond strength, ranging from 41% to 62%. This system proves particularly beneficial for crystalline marble, which shows the highest improvement under butterfly-type clips (61.63%). The moderate enhancement provided by the butterfly-type clip suggests that it serves as an effective option in applications where full mechanical engagement is essential, though not as extensively as the Z-type system.

The wire tie anchorage, on the other hand, yields the lowest improvement in bond strength, with increases ranging from 26% to 44%. This is likely due to the limited surface contact provided by the wire tie system, which results in a less effective mechanical engagement. Nonetheless, the incremental gains achieved by this system indicate its suitability in applications where more invasive or bulkier anchoring systems cannot be used.

In summary, the Z-type clip system results in the highest average bond strength increase of 82%, followed by 52% for the butterfly-type, and 35% for the wire tie. These results highlight the critical role of the anchorage system in optimizing shear bond strength, with the Z-type offering the most pronounced performance gains. In practical applications, the selection of an appropriate anchorage system, particularly the Z-type, could significantly enhance the durability and stability of stone cladding systems by improving both mechanical stability and adhesion.

#### 3.3.3. Effect of Curing Duration on Shear Bond Strength

**Figure 11:**
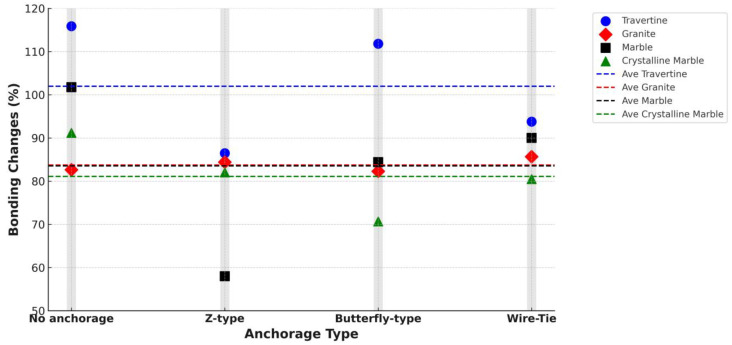
Bond strength changes (%) due to the increase in curing duration from 7 days to 28 days for various façade stones and anchorage types.

This section investigates the influence of curing duration—comparing the standard 7-day curing period with total 28-day curing—on the shear bond strength between façade stones and their concrete substrates. Although a 7-day curing period typically meets code requirements for mortar strength, this study includes a 28-day curing duration to provide deeper insights into the impact of prolonged curing on the adhesive bond. The goal is to quantify the bond strength improvement that can be achieved by extending the curing period, which can help structural designers optimize bonding performance in façade systems. Figure 11 illustrates the percentage increase in bond strength for the 28-day curing period compared to 7 days across different façade stones and anchorage systems. Overall, the bond strength shows a marked improvement across all conditions; however, the magnitude of the increase varies depending on the type of stone and anchorage used.

In specimens without any mechanical anchorage, the bond strength increased by 83–116%, highlighting the substantial effect of extended curing in conditions where the bond relies solely on adhesive attachment. This significant improvement suggests that in the absence of mechanical anchorage, extended curing is critical for enhancing adhesion between the stone and mortar, especially in stones like travertine, which exhibited the highest bond strength growth of 115.88%. For mechanically anchored specimens, butterfly-type clips and wire ties showed the highest bond strength improvements, with increases in the ranges of 71–112% and 81–94%, respectively. Z-type clips showed a more moderate improvement, with increases ranging from 58–86%, indicating that mechanical anchorage, particularly Z-type, benefits more from early curing stages, with less pronounced gains over extended curing periods.

On average, the bond strength improved by 98% in specimens without anchorage. In comparison, butterfly-type anchorage yielded an 87% increase, wire-type anchorage led to an 88% increase, and Z-type anchorage saw a 78% increase. These results underscore the importance of the curing period, particularly in non-anchored specimens, where prolonged curing has a much more significant effect on bond strength than in specimens with mechanical anchorage. Designers should pay particular attention to the curing duration when mechanical anchorage is absent, as it plays a crucial role in achieving optimal adhesion.

Travertine is more sensitive to curing, and by increasing the number of curing days to 28 days, about 101% higher bond strength was obtained compared to 7-day curing. Meanwhile, the rest of the stones experienced an increase in the range of 80 to 85 percent. Therefore, the result of this discussion for the construction industry can be that if travertine is used as a façade, increasing the number of curing days can have a higher effect on its bond strength to the substrate.

Interestingly, the improvement in compressive strength of the adhesive mortar between 7 and 28 days was notably lower than the improvement in bond strength. Compressive strength increased by 42.75%, 45.50%, 46.96%, and 53.93% for Ctrl, P1, P2, and P3, respectively, with an average increase of 47%. This indicates that while the adhesive mortar’s compressive strength continues to improve with curing, the bond strength between the mortar and the stone shows a much greater sensitivity to curing duration. Therefore, it can be concluded that curing has a more profound effect on bond strength than on compressive strength, particularly for stones with higher water absorption rates, where the adhesive attachment is more reliant on the curing process to achieve full bonding potential.

#### 3.3.4. Effect of the Polypropylene Dosages on Shear Bond Strength

**Figure 12:**
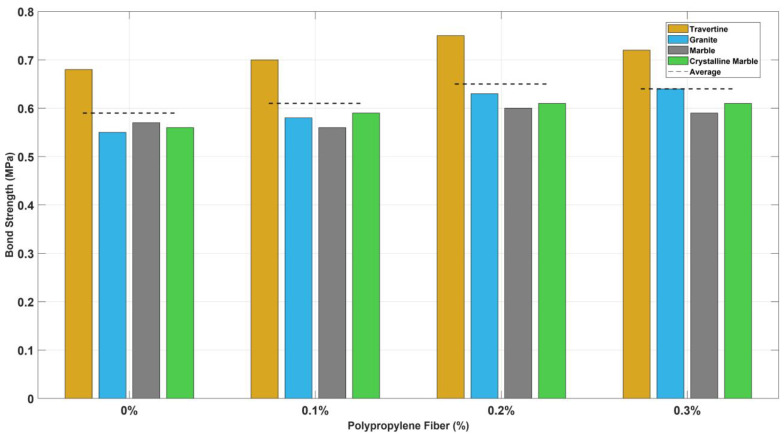
Shear bond strength for four façade stones across polypropylene fiber dosages averaged over all four anchorage conditions.

Figure 12 presents the effect of polypropylene fiber dosages on the shear bond strength of four façade stones, with results averaged across the four anchorage conditions: no anchorage, Z-type clips, butterfly-type clips, and wire ties. The inclusion of polypropylene fibers consistently enhanced the bond strength. The optimal fiber dosage was identified at 0.2% by volume, yielding an average bond strength of 0.65 MPa across all stone types, slightly outperforming the 0.3% dosage (0.64 MPa), suggesting diminishing returns beyond this point. The incorporation of polypropylene fibers into the adhesive mortar has been shown to significantly reduce shrinkage, thus enhancing bond strength. This reduction in shrinkage is attributed to the confining effect of the façade stone and the substrate concrete, which prevents the adhesive mortar between these layers from shrinking as it cures. Consequently, the presence of polypropylene fibers helps maintain structural integrity by minimizing shrinkage [17], ultimately reducing the potential loss of bond strength between the façade stone and the adhesive mortar.

Even a modest addition of 0.1% polypropylene fibers showed a clear improvement over the non-fibered mortar (P0.0), increasing the bond strength from 0.59 MPa to 0.61 MPa. This underscores the beneficial role of fiber reinforcement in adhesive performance. Notably, travertine consistently exhibited the highest bond strength across all polypropylene fiber dosages, outperforming the other stones in both non-fibered and fiber-reinforced mortars. This may be attributed to its higher porosity and the resultant increased mechanical interlock between the stone and the adhesive mortar. These findings underscore the importance of fiber dosage optimization, with 0.2% offering the best balance between enhanced bond strength and material efficiency.

The increase in bond strength with the incorporation of polypropylene fibers in the adhesive mortar is significant from an engineering perspective. This improvement is due to three main factors: (1) the reduction of shrinkage in the mortar, (2) the increase in tensile strength of the mortar, and (3) the bridging action of the fibers, which helps to slow the propagation of cracks under applied loads. From an economic standpoint, the use of polypropylene fibers is also advantageous, as they are relatively inexpensive. Adding polypropylene fibers to the adhesive mortar, particularly at the optimal dosage of 0.2%, has resulted in an approximately 10% improvement in bond strength between the façade and the mortar. This enhancement makes the use of polypropylene fibers a cost-effective solution for improving the performance of adhesive mortars.

### 3.4. Predicting the Bond Strength of the Façade Stones to Concrete Substrates

#### 3.4.1. Linear Regression Model

In this study, a linear regression model was formulated to predict the bond strength of façade stones to concrete substrates after 7 days of curing. The prediction was based on three key variables: the type of anchorage (AT), the water absorption capacity (WA) of the stone, and the volume percentage of polypropylene fibers (F) in the adhesive mortar. The developed regression is presented in Equation (1):

(1) BS=0.217+0.105(WA)+0.185(F)+0.11(AT)

where *BS* denotes 7-day bond strength (MPa), *WA* represents 24 h water absorption of façade stone (%), *F* refers to the percentage of polypropylene fibers in the adhesive mortar (as a volume percentage of concrete), and *AT* is the anchorage type, assigned a value of 1 for no anchorage, 2 for wire tie, 3 for the butterfly-type clip, and 4 for Z-type clip.

**Figure 13:**
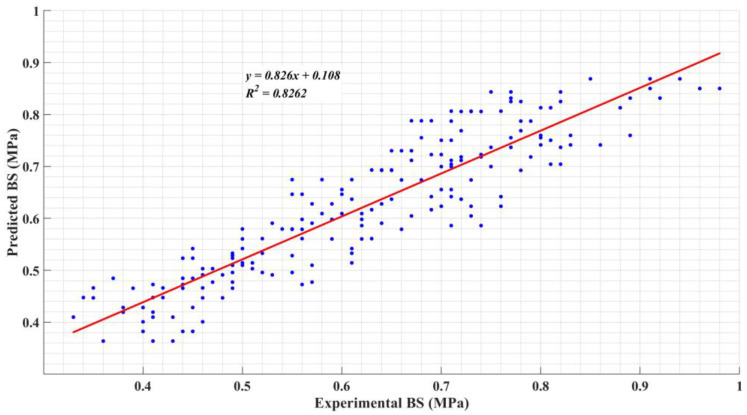
Linear regression predictions compared with experimental results.

The model’s accuracy was evaluated by comparing predicted values to experimental results, as shown in Figure 13. The R^2^ value, a key indicator of the model’s fit, was found to be 0.8262, suggesting a reasonably strong correlation between the predicted and actual bond strength values.

While the model offers valuable insights into the linear relationships between bond strength and the three variables, the complexity of the interactions between these factors—particularly with respect to non-linear behavior—may limit the regression’s predictive capability. The inclusion of a single linear term for each variable, although useful, may not fully capture the intricate dependencies between anchorage type, fiber content, and the stone’s absorption characteristics. Nonetheless, the linear model provides an initial framework for understanding how these factors influence bond strength, and it serves as a benchmark against which more advanced predictive techniques, such as fuzzy logic systems, can be compared.

#### 3.4.2. Overview of Generalized Mamdani’s Fuzzy System

The fuzzy system applied in this study uses a structured three-step algorithm for the prediction of bond strength. It begins by assuming that N input–output pairs $\left(x^{N},y^{N}\right)$ are given, where $x^{k}=(x_{1}^{k},\ldots ,x_{n}^{k})$, ∀k∈{1,…,N}, i.e., $X^{K}\in U=U_{1\times }U_{2\times \ldots \times }U_{n}\subseteq R^{n}$ and $\gamma^{Κ}\epsilon \;V\subseteq R$ are provided as the dataset. For each input $U_{j}$ (j=1,…,n), M fuzzy sets are defined $A_{j}^{l}$ (l=1,…,M), and it is essential that these fuzzy sets be complete within the input space $U_{j}$. Specifically, this condition ensures that for any given $x_{j}\in U_{j}$, an $A_{j}^{l}$ there exists such that $\mu_{A_{j}^{l}}(x_{j})\neq 0$.

As an example, consider the fuzzy set $\mu_{A_{j}^{l}}(x_{j})$ defined by a Gaussian membership function, which can be expressed as in Equation (2); it may be considered that $\sigma_{j}^{l}=\left|{\bar{x}}_{j}^{\left(l+1\right)}-{\bar{x}}_{j}^{l}\right|/2$ (l=1,2,…,M):

(2) $$\mu_{A_{\;j}^{\;l}}(x_{j})=e^{-\;\;{\left(\frac{x_{j}-{\bar{x}}_{\;j}^{\;l}}{\sigma_{j}^{l}}\right)}^{2}}$$

where ${\bar{x}}_{j}^{l}$ denotes the center of the fuzzy set $A_{j}^{l}$, and $\sigma_{j}^{l}>0$. Using these fuzzy sets, the algorithm constructs the fuzzy system following Equation (3):

(3) $$f(x)=\frac{\sum_{l=1}^{M}{\bar{y}}^{l}T_{\alpha }(\mu_{A_{1}^{l}}(x_{1}),\ldots ,\mu_{A_{n}^{l}}(x_{n}))}{\sum_{l=1}^{M}T_{\alpha }(\mu_{A_{1}^{l}}(x_{1}),\ldots ,\mu_{A_{n}^{l}}(x_{n}))}$$

where $x\in U\subseteq R^{n}$ is the input to the fuzzy system; $f\left(x\right)\in V\subseteq R$ represents the output of the fuzzy system, which serves as the desired approximation; the term $T_{\alpha }$ is derived using the Dombi family of t-norms (λ>0), as defined in Equation (4):

(4) $$T_{\lambda }(x,y)=\left\{\begin{array}{l} 0\;ifx=0\;or\;y=0\\ \frac{1}{1+{\left({\left(\frac{1-x}{x}\right)}^{\lambda }+{\left(\frac{1-y}{y}\right)}^{\lambda }\right)}^{\frac{1}{\lambda }}}\;otherwise \end{array}\right.$$

In this system, λ controls the smoothness of the t-norm, allowing for flexible adjustments in system behavior based on the desired accuracy and sensitivity.

The inference engine of this generalized Mamdani fuzzy system is based on Equation (3) and uses a singleton fuzzifier and a center-average defuzzifier, as described by Equations (5) and (6):

(5) $$\mu_{A^{’}}(t)=\left\{\begin{array}{cc} 1 & t=x\\ 0 & otherwise \end{array}\right.$$

(6) $$y^{*}=\frac{\sum_{l=1}^{M}{\bar{y}}^{l}w_{l}}{\sum_{l=1}^{M}w_{l}}$$

where $y^{*}\in U$; ${\bar{y}}^{l}$ is the center of the l’s individual output fuzzy set ${\bar{B}}^{l}$, and $w_{l}$ represents the fuzzy weight of each rule.

#### 3.4.3. Prediction Using the Fuzzy Logic System

**Figure 14:**
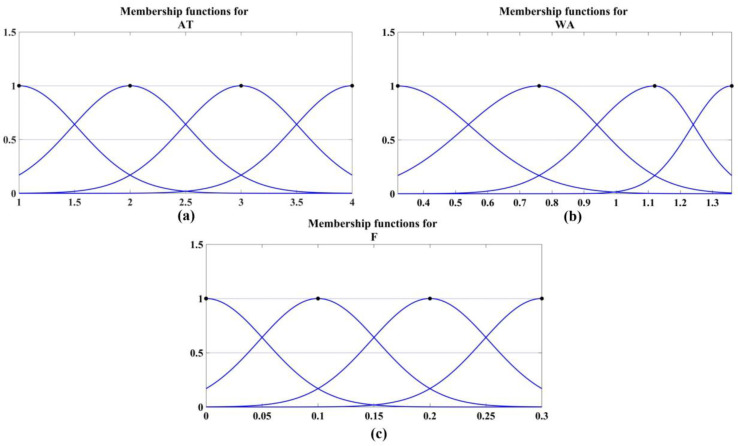
Membership functions for three inputs: (**a**) anchorage type, (**b**) water absorption of the façade stone, and (**c**) volume percentage of polypropylene fiber.

The fuzzy logic system, with its generalized Mamdani inference engine and Dombi t-norms, was applied to predict the bond strength between façade stones and their concrete substrates. Three input variables were considered: anchorage type, water absorption of the façade stone, and the volume percentage of polypropylene fibers in the adhesive mortar. Gaussian membership functions were defined for these inputs, as shown in Figure 14.

**Figure 15:**
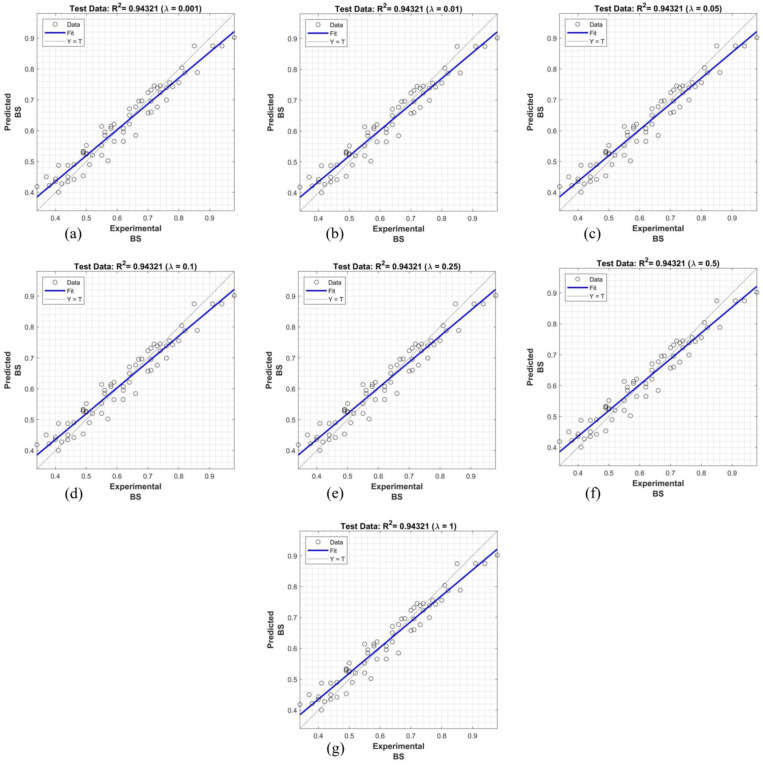
Prediction of bond strength with proposed fuzzy logic inference system using Dombi family of t-norms: (**a**) λ = 0.001, (**b**) λ = 0.01, (**c**) λ = 0.05, (**d**) λ = 0.1, (**e**) λ = 0.25, (**f**) λ = 0.5, and (**g**) λ = 1.

**Figure 16:**
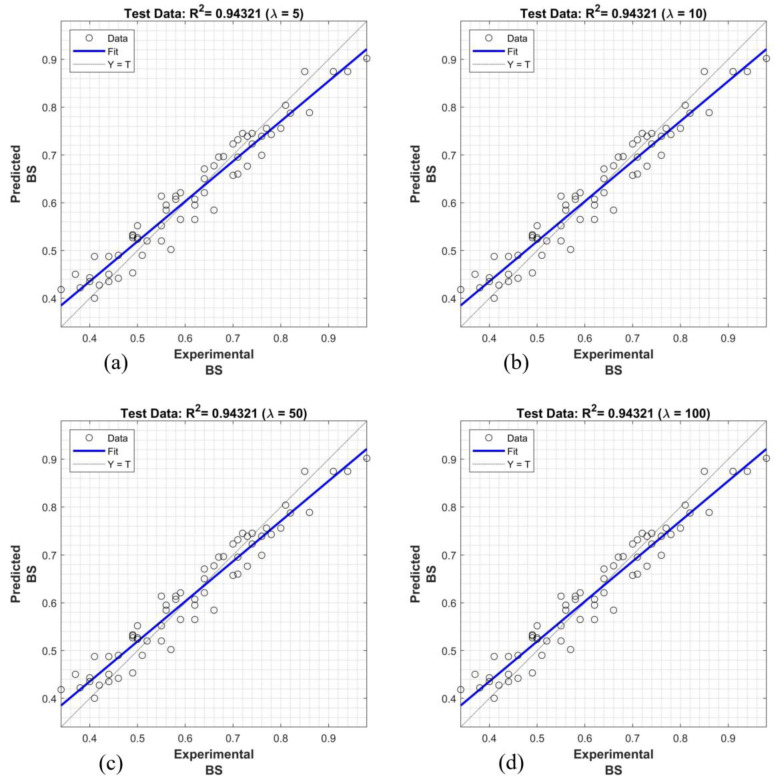
Prediction of bond strength with proposed fuzzy logic inference system using Dombi family of t-norms: (**a**) λ = 5, (**b**) λ = 10, (**c**) λ = 50, and (**d**) λ = 100.

**Figure 17:**
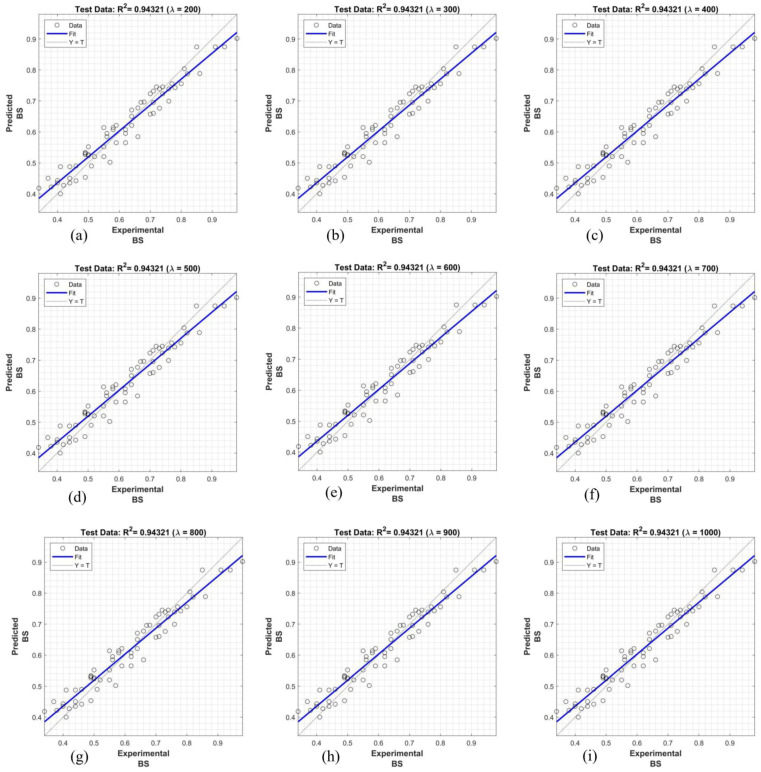
Prediction of bond strength with proposed fuzzy logic inference system using Dombi family of t-norms: (**a**) λ = 200, (**b**) λ = 300, (**c**) λ = 400, (**d**) λ = 500, (**e**) λ = 600, (**f**) λ = 700, (**g**) λ = 800, (**h**) λ = 900, and (**i**) λ = 1000.

The findings presented in Figure 15, Figure 16 and Figure 17 demonstrate the superior performance of the proposed system using the Dombi family of t-norms in predicting bond strength, with R^2^ values ranging from 0.8697 to 0.9584. These results exhibit a significant improvement over the linear regression model. Notably, the system achieved the highest predictive accuracy for the configurations T_600_, T_700_, T_800_, T_900_, and T_1000_, reaching an R^2^ value of 0.9584. In general, the fuzzy systems employing λ ≤ 1 and λ ≥ 600 consistently provided more accurate bond strength predictions between the façade stone and its substrate compared to other λ values.

The superior performance of the proposed fuzzy system, particularly using the Dombi family of t-norms, for predicting bond strength is significantly influenced by the parameter λ. The findings indicate that fuzzy systems with λ ≤ 1 and λ ≥ 600 consistently yield more accurate predictions compared to other λ values. When λ is small, the t-norm behaves conservatively, emphasizing lower membership values and producing reliable outputs in uncertain conditions. Conversely, larger λ values generate sharper, more confident predictions, capturing intricate relationships between input variables like anchorage type, fiber dosage, and water absorption. This balance between conservative and confident outputs likely explains the system’s superior accuracy for λ within these ranges.

This approach confirms that fuzzy logic systems incorporating a generalized Mamdani’s inference engine and Dombi t-norms can serve as an effective predictive tool for complex material behaviors in engineering applications. By accounting for non-linearities, the fuzzy model demonstrates superior accuracy compared to linear regression techniques.

As a final consideration in this study, the accuracy of the prediction model is of particular importance. While a difference of 0.1 to 0.2 MPa may not be critical in many engineering contexts, in this study, the sensitivity of the issue must be taken into account. The potential for stone detachment from the façade poses a significant risk, leading to potential financial losses and safety hazards. In some cases, a difference of 0.2 MPa can represent approximately 30% of the bonding strength between the façade stone and the adhesive mortar. Thus, achieving a precise prediction of bond strength is crucial to ensure the stability and safety of the façade system.

## 4. Conclusions

This study comprehensively evaluated the bond strength between four prominent façade stones—travertine, granite, marble, and crystalline marble—and their concrete substrates. The investigation focused on three critical factors: curing duration, polypropylene fiber dosages in the adhesive mortar, and the type of mechanical anchorage. Through a series of controlled experiments, the research aimed to establish a deeper understanding of the shear bond strength under varying conditions, contributing valuable insights to enhance the durability and performance of stone-clad façades. Importantly, the study introduced a novel predictive model based on fuzzy logic systems, providing a more accurate approach to estimating bond strength compared to traditional linear regression methods.

The results demonstrate that among the mechanical anchorage systems tested, Z-type anchorage produced the highest bond strength, followed by butterfly-type and wire tie anchorages. In cases without mechanical anchorage, extended curing periods showed a substantial impact on bond strength, particularly for travertine, which exhibited heightened sensitivity to curing duration. Polypropylene fiber addition at an optimal dosage of 0.2% by volume led to the highest bond strength, although all tested dosages, including 0.1%, 0.2%, and 0.3%, contributed positively to the adhesive performance. Furthermore, the findings suggest a trend where higher water absorption correlates with improved bond strength, albeit in a non-linear fashion, with travertine consistently outperforming the other stones.

The development of a fuzzy logic prediction model using the Dombi family of t-norms represents a significant advancement in the field. This system yielded more accurate predictions of bond strength than linear regression, with an R^2^ up to 0.9584. These findings underscore the versatility of the fuzzy logic approach in addressing the complexities of façade stone bonding. However, the study’s scope was limited to the evaluation of polypropylene fibers, specific anchor types, and stone varieties. The absence of detailed microscopic analysis represents an area where further exploration could enhance understanding of material properties at a fundamental level. Future research should explore a broader range of fibers, mix proportions, alternative anchorage systems, and additional stone types to further generalize the findings and refine predictive models.

## Figures and Tables

**Table 1:** Properties of polypropylene fibers.

Property	Value
Length	6 mm
Diameter	200 μm
Density	0.91 g/cm^3^
Appearance	Single strand
Melting Temperature	160 °C
Water Absorption	0%
Chemical Formula	(C_3_H_6_)_n_
Source	Virgin-derived

**Table 2:** Mix proportions of the adhesive mortars.

ID	Cement (kg/m^3^)	Polypropylene Fibre (% Vm)	W/C	Fine Aggregate (kg/m^3^)
Ctrl	550	0	0.5	1437
P1	550	0.1	0.5	1437
P2	550	0.2	0.5	1437
P3	550	0.3	0.5	1437

**Table 3:** Properties of galvanized steel used for anchorage.

Yield Stress (MPa)	Ultimate Stress (MPa)	Ultimate Strain	Modulus of Elasticity (GPa)
235	370	0.15	200

**Table 4:** Results for 24 h water absorption of stones.

Façade Stone	Travertine	Granite	Marble	Crystalline Marble
Water absorption (%)	1.36%	0.32%	1.12%	0.76%

**Table 5:** Compressive and tensile strength of the adhesive mortars.

Mix Proportion		Ctrl	P1	P2	P3
Compressive strength (MPa)					
Curing Process	7-day	17.43	17.72	18.05	16.42
	28-day	24.88	25.79	26.52	25.28
Tensile strength (MPa)					
Curing Process	7-day	1.57	2.18	2.58	2.15
	28-day	2.21	3.12	3.86	3.23

## Data Availability

The original contributions presented in the study are included in the article, further inquiries can be directed to the corresponding author.
